# The Trunk of Henle and Belcher’s Vein: Important Venous Landmarks in Robot-Assisted Pancreatoduodenectomy

**DOI:** 10.3390/jcm14176144

**Published:** 2025-08-30

**Authors:** Ernesto Barzola, Jordi Navinés-López, Alessandro M. Bonomi, Miguel Ángel Gómez-Bravo, Esteban Cugat, Marc G. Besselink

**Affiliations:** 1 H. Clinic Barcelona, Faculty of Medicine, University of Barcelona, 08036 Barcelona, Spain; ejbarzola@clinic.cat; 2 HBP Unit, Department of General and Digestive Surgery, Hospital Universitari Germans Trias i Pujol de Badalona, 08916 Barcelona, Spain; esteban.cugat@gmail.com; 3 General Surgery Unit ASST-Brianza, Vimercate Hospital, 20871 Vimercate, Italy; alessandro.bonomi@unimi.it; 4 HBP and Liver Transplant Unit, Department of General and Digestive Surgery, University Hospital Virgen del Rocio, 41013 Sevilla, Spain; miagbravo@gmail.com; 5 Department of Medical Sciences, Faculty of Medicine, University of Seville, 41004 Sevilla, Spain; 6 Department of Surgery, Location University of Amsterdam, Amsterdam University Medical Center, 1105 Amsterdam, The Netherlands; m.g.besselink@amsterdamumc.nl; 7 Cancer Center Amsterdam, 1105 Amsterdam, The Netherlands

**Keywords:** robot-assisted pancreatoduodenectomy, Trunk of Henle, pancreatic surgery

## Abstract

**Background:** The Trunk of Henle and the posterosuperior pancreaticoduodenal vein (Belcher’s Vein) are consistent anatomical landmarks of the portomesenteric venous system. Their recognition is particularly relevant in robot-assisted pancreatoduodenectomy (RAPD), where uncinate process dissection from the portal–mesenteric axis represents the most technically demanding step. **Methods:** We describe a stepwise robotic surgical approach emphasizing the identification, isolation, and safe division of the Trunk of Henle and Belcher’s Vein. Intraoperative illustrations are provided to demonstrate the use of these veins as reproducible landmarks during dissection of the pancreatic head and uncinate process. **Results:** Incorporating these veins as key reference points facilitates precise dissection, improves vascular control, and minimizes intraoperative bleeding. Their consistent anatomical presence allows systematization of the uncinate process approach and reliable exposure of the portal–mesenteric axis. **Conclusions:** The Trunk of Henle and Belcher’s Vein serve as valuable venous landmarks in RAPD. Their routine identification may improve surgical safety, reduce conversion risk, and contribute to a standardized, reproducible methodology for robotic pancreatic head resection.

## 1. Introduction

Robot-assisted pancreatoduodenectomy (RAPD) is a complex procedure because of the necessity of carrying out dissection near major fragile vascular structures with multiple potential anatomical variants [1], which may lead to significant morbidity if misinterpreted. Notwithstanding, the oncological outcomes of RAPD have improved in recent years, despite its long learning curve, without a negative effect on 90-day mortality [2]. However, the robotic approach has helped to overcome some of the challenges linked to the laparoscopic approach, not only by improving precision and exposure, but also because of the maneuverability of the robotic wristed tooltips and the system’s improved three-dimensional vision, overall simplifying the surgical movements [1,2,3,4].

The uncinate process is formed after the embryological fusion of the ventral pancreatic bud over the dorsal pancreatic bud, and is named after its hook-like appearance. It projects from the inferior aspect of the head of the pancreas and extends behind the superior mesenteric vessels, and it lies in extremely close proximity to the superior mesenteric vein and often the artery, separated from them by a thin fat plane; thus, it has become the resectability cornerstone of surgical treatment for pancreatic cancer.

Anatomically, the portal–mesenteric axis includes the superior mesenteric vein and the portal vein (PV), with their confluence with the splenic vein being a critical part of the portal venous system. The superior mesenteric vein (SMV) and its arterial counterpart, the superior mesenteric artery (SMA), have a complex three-dimensional anatomy with variable vascular patterns [5], which may pose challenges for oncological resection.

Gaining an accurate understanding of the surgical anatomy, as well as properly identifying all the related vessels, are of paramount importance during a RAPD. Specifically, the Trunk of Henle and Belcher’s Vein are the two main landmarks to consider when interpreting this anatomy, due to their anatomical relevance and consistency.

On the one hand, the Trunk of Henle (TH) is a frequent collateral in the portal venous system. Its structure can vary greatly, but its most typical form is the gastro-pancreato-colic trunk, connecting the right gastro-pancreato-colic vein and the middle colic vein with the SMV [6].

On the other hand, described by Cameron and Belcher as the posterosuperior pancreaticoduodenal vein, Belcher’s Vein (BV) forms part of the venous drainage of the head of the pancreas and is a major tributary to the PV, which highlights the importance of its identification during the uncinate process dissection stage of the Whipple procedure. This vein was named after one of Cameron’s assistants: “ironically due to its description of being short, fat, and always getting in the way” [7].

The TH and BV are reliable venous landmarks that may be used for referential assistance during robotic procedures in order to improve the safety of navigating the pancreas head and uncinate process region. Identifying and controlling these veins may be essential for the safe mobilization of the pancreatic head and uncinate process from the PV. This technical note describes a standardized stepwise methodology for identifying and controlling the Trunk of Henle and Belcher’s Vein during RAPD, highlighting their role as reliable venous landmarks for safer and more reproducible resections.

## 2. Surgical Resection Technique

### 2.1. Patient Selection and Positioning

**Figure 1:**
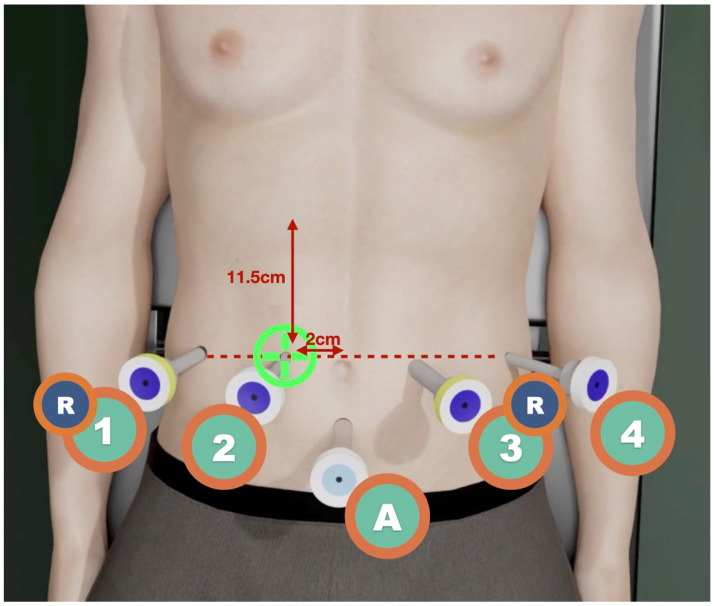
Trocar placement during RAPD. T2 is marked 11.5 cm from the costal margin and 2 cm to the right of the umbilicus; T3 and T4 are aligned horizontally under direct vision, with T1 placed slightly cranially. An assistant port (A) is placed 7 cm equidistantly from T2 and T3.

A patient is considered suitable for the robotic approach if they have a BMI of <35 kg/m^2^. The patient is placed 10° in the reverse Trendelenburg position. The robot arm is docked to the right of the patient. A closed 12 mm Hg pneumoperitoneum is achieved with a Veress needle placed at Palmer’s point. Robotic trocar placement follows a straight line from right to left, as shown in Figure 1. The length of each incision is indicated by marking the skin with a trocar scabbard after insufflation has been completed. Trocars are placed sequentially: starting from T2, one is placed 11.5 cm from the mid-costal margin and 2 cm to the right of the patient’s umbilicus; then, the others are placed under laparoscopic direct vision, from T1 (slightly more cranial) to T4, using reducing (R) cannulas for T1 and T3, to form a horizontal line. One or two laparoscopic ports for the tableside surgeon may be placed 7 cm equilaterally down from T3R and T4.

### 2.2. Stepwise Dissection Approach

The dissection begins by dividing the gastrocolic ligament to access the lesser sac, proceeding laterally to the short gastric vessels on the left, and medially to expose the first part of the duodenum to the right. Alternatively, the first step can be Treitz dissection.

The right colonic flexure is widely mobilized to expose the duodenum, SMV, and pancreatic head, followed by a complete Kocher maneuver to detach the pancreatic head from the retroperitoneum, down to the left side of the aorta, to retrieve the interaortocaval lymph nodes and expose the left renal vein and the origin of the SMA.

The proximal jejunum is exposed to the right side of the transverse colon and the first jejunal loop by dividing it with a 60 mm white reload robotic Endostapler and dissecting it straight to the mesenteric root, taking the Treitz ligament as well. The specimen is then rotated 270° clockwise and uncrossed posteriorly to the mesenteric root to retrieve it from the upper-right quadrant. This maneuver mobilizes the first jejunal loop and the third and fourth portions of the duodenum by transposing the duodenum and proximal jejunum to the right of the SMV–SMA axis in the coronal plane. This positioning is key for the safe and effective dissection of the uncinate process and the SMV.

Depending on the type of robotic approach, whether robotic-assisted or fully robotic [3] (defined as the complete use of the robotic technique for PD, including resection and reconstruction, without laparoscopic or hand-assisted techniques, but including the use of laparoscopic ports by a tableside surgeon, which is regarded as standard), hilar lymphadenectomy may be performed prior to or after the duodenal uncrossing. In both approaches, the final resective step of the RAPD is the dissection of the uncinate process from the SMV, once the specimen has been collected from the upper-right quadrant, in order to take full control of the PV/SMV vascular axis, as a key milestone before starting to spare the uncinate process.

Once the minor omentum above it has been opened, the posterior wall of the stomach is dissected, and the right gastric artery and the right gastroepiploic vessels are transected with locked clips and a vessel sealer. The antrum of the stomach is divided with one or two 60 mm blue reload robotic Endostaplers, placed just upstream of the pylorus, to improve access to the pancreas and allow exposure of the suprapancreatic lymph nodes and hepatic hilum. Then, the right gastric artery is returned to its origin, and lymphadenectomy is performed from level 8A upwards to the hilar plate.

The gastroduodenal artery (GDA) is then dissected, and the vessel loop is surrounded, clipped, and ligated with 2/0 silk prior to its mechanical transection with a 30 mm white reload robotic Endostapler for secure vascular control, leading to the exposition of the common, proper, right, and left hepatic arteries and the bile duct.

Although not considered the default technique in RAPD, pylorus-preserving pancreatoduodenectomy has also been described. In this case, the right gastroepiploic arcade must be identified and preserved to maintain adequate blood supply to the pylorus [1].

Once the superior and inferior limits of the pancreas neck have been dissected, a tunnel is created between the posterior surface of the pancreatic neck and the anterior aspect of the PV. After placing a loop around the pancreas neck, the pancreas isthmus is incised with monopolar curved scissors, and the main pancreatic duct is identified and divided with a ‘cold cut’ to prevent occlusion or stenosis of the pancreatic duct.

Once the duodenum specimen has been successfully uncrossed on the right side, a slight countertraction using robot arm 1 next to the gastroepiploic locked clip on the specimen side facilitates safe dissection of the uncinate process by simplifying the torsional position between the duodenum and the SMA/SMV axis. In this step, it is essential to properly identify and manage the two major key venous landmarks.

### 2.3. Trunk of Henle (TH)

**Figure 2:**
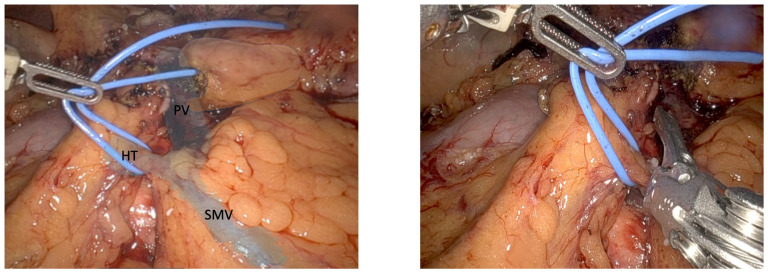
Intraoperative view during uncinate process resection. The Trunk of Henle (HT) is isolated using a vessel loop for vascular control and divided using a locking clip.

The TH is found in 86.9% of people [6]. It is usually found at the lower border of the pancreas, close to the pancreatic head, and extends up to 20 mm downward along the front-right side of the SMV [7,8], draining straight into it. The average diameter of the trunk is 4.2 mm (see Figure 2). The most frequent branches from the pancreas and colon are the anterior superior pancreaticoduodenal vein, seen in 88.3% of patients, and the superior right colonic vein, seen in 82.5% of patients [6]. Identifying this structure and performing careful monopolar scissor dissection are important for obtaining enough vascular stumps before looping it, in order to obtain enough space to place the 30 mm white reload robotic Endowrist^®^ (Intuitive Surgical, Sunnyvale, CA, USA) mechanical stapler or locking clips, depending on the structure’s width. This step is greatly facilitated by countertraction of robot arm 1 next to the locking clip of the gastroepiploic vessels, creating some tension and allowing better visualization of the TH to ensure good surgical results.

### 2.4. Belcher’s Vein (BV)

**Figure 3:**
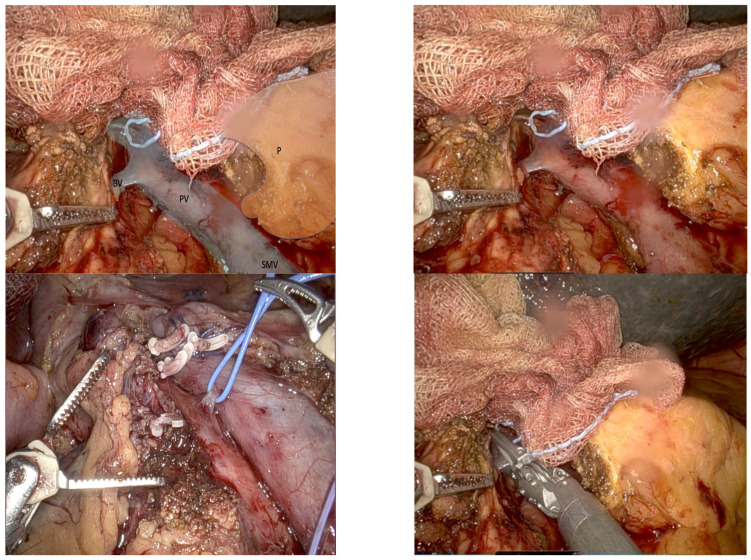
The posterosuperior pancreaticoduodenal vein, also referred to as Belcher’s Vein, is isolated using a vessel loop. A polymer locking clip is applied to achieve vascular control. The portal vein (PV), superior mesenteric vein (SMV), and Belcher’s Vein (BV) and P (Pancreas)are identified.

Identified during the pancreatic head dissection, this vein is a consistent main tributary of the PV, making it a major landmark for dissection of the uncinate process. BV is present in 90% of patients [7,9,10]. It joins the right posterolateral wall of the PV, usually within 1 cm from the splenic vein–portal vein confluence [7]. BV transection should be performed using polymer locking or a metal clip (see Figure 3), allowing the broad and safe exposure of the PV and SMV and thereby opening the posterior hilar portal confluence, helping to complete the 360° PV dissection, before ending the pancreas head mobilization. Ideally, BV transection should be postponed until all SMA branches have been transected to prevent venous congestion in the pancreatic head.

After completing the PV dissection, the common bile duct (CBD) is isolated upstream and clamped proximally to the cystic duct with a bulldog, before being transected with monopolar scissors. The gallbladder and the remaining surgical Whipple specimen are taken in bloc, removed via Pfannenstiel incision, and placed in a specimen retrieval bag.

## 3. Surgical Anatomy

The dissection of the uncinate process is known to be the most technically challenging step in the minimally invasive approach to surgery on the pancreas head, due to the small and posterior collaterals arising from the SMV. During resection of the uncinate process, the correct exposure of the SMV is essential [11]. The dissection proceeds from caudal to cranial, along the SMV-PV axis. Small venous branches from the PV, SMV, and SMA must be carefully identified, dissected, and divided, but the risk of bleeding from variant collaterals remains significant [12]. Especially for SMA branches, clips are advised. Although it is unclear what factors are related to the lower rates of conversion to open surgery seen with RAPD versus with the laparoscopic approach [13], it is reasonable to argue that the major advantage of the seven degrees of movement of the robotic tooltips may be a determinant of successful vascular control and the avoidance of non-programmed bleeding-related conversions.

Accurate identification and secure vascular control contribute to a standardized and reproducible process of dissection during RAPD. Thus, it is essential to know the anatomical variations in the SMA/SMV collaterals. A comprehensive description of these structures is essential for surgical planning and intraoperative navigation. Nevertheless, standard surgical textbooks often lack a detailed description of the tributaries of the SMV and PV [7,8], which are paramount for the safe planning and performance of a RAPD [7,8,9].

Several veins, particularly those draining the uncinate process and proximal jejunum, require careful dissection and ligation. In some cases, ligation may be required to achieve adequate exposure and control of the SMV and its tributaries, or to manage bleeding risks [11,14], avoiding small vein wall tears as the dissection progresses. This is why the duodenojejunal uncrossing maneuver allows safer and more direct access to the key vascular structures of the SMV-PV axis, including the TH, the inferior pancreaticoduodenal vein, and the remaining small pancreatic tributaries [15,16,17,18]. Thus, preserving the FJV/JT ensures safe dissection around the SMA/SMV axis from the mesenterium root [14,17,19].

**Figure 4:**
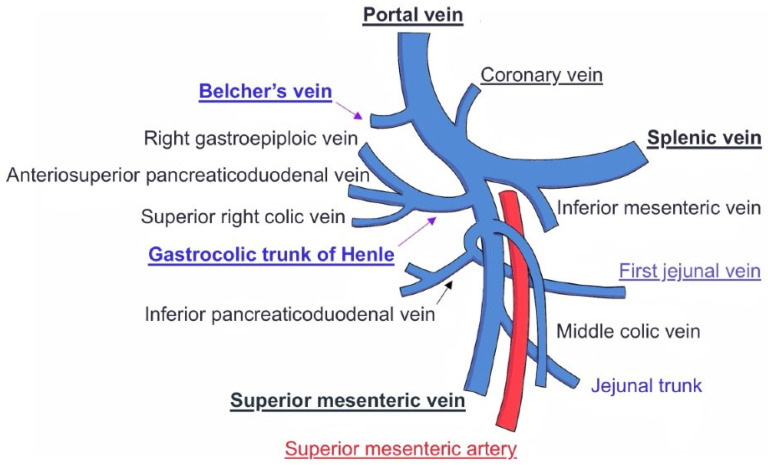
Key tributaries of the portal vein, highlighting Belcher’s Vein and the Trunk of Henle. Conceptual design modified from Desai et al. [15]. Art: E.B., J.N.L.

On the one hand, the TH must be differentiated from the first jejunal vein. Despite frequent anatomical variations in the venous outflow of the uppermost jejunum, a common configuration, known as the jejunal trunk (JT), may occur when a shared trunk arises from the confluence of the inferior pancreaticoduodenal vein and the first jejunal vein (FJV), or first jejunal trunk (FJT), when both the FJV and the second jejunal vein (SJV) join to drain this territory [15]. The FJV is a major PV tributary draining the proximal jejunum, and often receiving the inferior pancreaticoduodenal vein. The FJV/JT is a major vein draining into the portal system, running close to the SMA [7]. It maintains venous drainage of the proximal jejunum and often lies posterior to it [5,15]. The FJV/JT typically receives tributaries from the inferior pancreaticoduodenal vein, which serves as the primary drainage vein for the uncinate process [6]. Moreover, the FJV/JT may course either above or below the TH, and may vary its position relative to the SMA: anterior in 20–26% of cases and posterior in 71.8–80% of cases [5,6,9,13] (see Figure 4). Since the JT itself should not be cut during the Whipple procedure, it is crucial to differentiate it from the TH.

On the other hand, the BV is a valuable anatomical landmark during the final step of dissection of the uncinate process (see Figure 3). Once the TH has been taken, the uncinate process begins to detach from the SMV-PV axis. As BV arises in a posterolateral position, it may be unintentionally injured during dissection if the surgeon is not aware of it. This is why its proper identification and control enables the final step of the uncinate process dissection to be performed without significant incidental bleeding, but at the same time, allows the possibility of dissecting the PV in 360° and completing the radical hilar lymphadenectomy with precision and vascular control.

## 4. Tips and Tricks

The TH and BV are valuable landmarks for guiding dissection of the PV. Their identification allows for the maintenance of a bloodless field during the dissection, leading to a safer resection plane and the achievement of wide exposure of the SMV and PV along the SMV/PV axis [11,14].

The specific approach may vary depending on the chosen dissection tool. The PV dissection plane may be entered either with harmonic shears through the accessory port, or with a vessel sealer or monopolar scissors under the purely robotic approach.

The SMA/PV axis is approached caudocranially from the mesenterium root, starting from the dissection plane obtained after mobilization of the right colon. It is recommended that this mobilization be wide enough to avoid transverse tearing that may transpose the TH’s branches horizontally, what may complicate the identification of its root. The middle colic vein may be followed upwards to uncover the SMA/PV surgical plane. The right colic vein may be used as well to determine the draining level of the TH. Sometimes the middle colic vein itself may drain into the TH. If so, it may be recommended to preserve it before severing the TH.

The BV root is usually uncovered during dissection of the mid-uncinate process, once the TH has been already taken. The direction of its drainage into the PV is slightly posterior and caudal, which underlines the importance of it being inspected before starting its dissection.

Once the main vascular structure has been determined, fine dissection is mandatory. Usually, this is achieved by careful gentle cold dissection with closed monopolar scissors, strictly following the adventitious layer, and avoiding direct contact with the vascular wall. This allows the surgeon to obtain enough of a vascular stump to apply either clips or a stapler.

The placement of a vessel loop is not needed, because the control position may become lost during the necessary maneuver, and even this does not happen, it may tear apart the vein, resulting in risky management with no technical benefit.

The TH and VB can usually be controlled using locking clips or a stapler, depending on the diameter of the vein. While the VB may be controlled routinely with clips, the TH allows for the placement of a stapler, due to its position in alignment with the T3 trocar and with enough uncovered vascular wall left to place the 30 mm white stapler, under the surgeon’s sight, from grip to tip.

Importantly, the routine use of staplers is not mandatory; effective hemostasis can be achieved using a polymer locked clip for the TH and a metal clip for Belcher’s Vein, in combination with vessel sealer energy devices. If a stapler is placed with its end not directly visible, this may result in inadverted lesions.

## 5. Discussion

As robotic surgery improves the visibility and precision of dissection, more effective handling of the TH, FJV/JT, and BV can be achieved, not only improving the safety of dissection and preventing unexpected vascular lesions that may heighten the risk of conversion, but also leading to a more structured and systematized dissection compared with open or laparoscopic approaches, which, in turn, also improves the safety of dissection.

Expertise and common sense regarding surgical fine dissection has led to the identification of two key landmarks during resection of the uncinate process, enforcing perspectives taken in papers on the classical open approach and emphasizing the value of Belcher’s Vein [7] not merely as a relevant anatomical structure, but also as a valuable landmark in the robotic approach. These findings support those of previous anatomical studies and emphasize the importance of performing RAPD using these venous landmarks [18].

The dissection of the uncinate process from the PV is commonly a necessary final step in the resection phase of a RAPD, regardless of the approach used (either robot-assisted or purely robotic). This is why the proper identification and control of such key structures is paramount in order to reduce the risk of bleeding, thus limiting bloodshed in the surgical site in the critical final step of the procedure, while at the same time reducing the risk of conversion just before the reconstruction phase of the procedure.

As such, the identification of these two structures as truly useful anatomical landmarks during RAPD opens the path to refining robotic methodology, which, in turn, constitutes a methodological basis for systematization of the procedure.

Systematization of the robotic procedure is essential in order to ensure comparable and reproducible results, improving the oncological outcomes associated with the robot-assisted minimally invasive approach to resection of the pancreatic head and uncinate process.

## 6. Conclusions

The TH and BV are valuable anatomical landmarks during RAPD. Their identification and proper dissection are useful for guiding dissection of the uncinate process from the PV, thus refining the stepwise approach of the final resection step of RAPD. Their use as key referential landmarks may promote good vascular control, as well as refining the robotic methodology, contributing to its systematization.

## Data Availability

The datasets generated during the current study are available from the corresponding author upon request. All data generated or analyzed in this study are included in this published article.
